# Applications of Artificial Intelligence (AI) for Diagnosis of Periodontal/Peri‐Implant Diseases: A Narrative Review

**DOI:** 10.1111/joor.14045

**Published:** 2025-06-04

**Authors:** Rupanjan Roy, Aditi Chopra, Shaswata Karmakar, Subraya Giliyar Bhat

**Affiliations:** ^1^ Department of Periodontology Manipal College of Dental Sciences, Manipal, Manipal Academy of Higher Education, Manipal Karnataka India; ^2^ Department of Preventive Dental Sciences, Division of Periodontology, College of Dental Surgery Iman Abdulrahman Bin Faizal University Dammam Kingdom of Saudi Arabia

**Keywords:** artificial intelligence, artificial neural network (ANN), implants, machine learning, periodontal diagnosis, periodontal disease

## Abstract

**Background:**

Artificial intelligence (AI) and various subunits of AI such as artificial neural networks (ANN), Convolutional neural networks (CNNs), machine learning (ML), deep learning (DL) and deep neural networks (DNN) are being tried to diagnose and plan treatment for periodontal diseases.

**Aim:**

This narrative review aims to discuss the current evidence on the applications of AI for the diagnosis and risk prediction of periodontal/peri‐implant diseases.

**Method:**

A search strategy with the following keywords: (Artificial intelligence [MeSH Terms]) AND (Periodontal disease [MeSH Terms]) was used to search for articles from 2000 to 2024.

**Results:**

AI models using patient‐related data, signs and symptoms of the disease, immunological biomarkers and microbial profiles aid in effective diagnosis and planning treatment. AI is also used in periodontal diagnosis of pathological and anatomical landmarks such as cementoenamel junction, bone levels, furcation defects, nature and system of dental implants placed, degree of implant or tooth fractures and periapical pathology, assessing the severity and grading of periodontal or peri‐implant disease/conditions, assessing the signs and symptoms of periodontal/peri‐implant disease and determining the prognosis of implant and periodontal treatment. Studies have compared the diagnosis made by dentists and AI‐based models and found AI models to be more effective and quicker in diagnosis than dentists.

**Discussion:**

AI‐based tools such as DL, ML, CNN, and ANN are more effective and quicker for timely diagnosis, risk assessment, and treatment plans for periodontal and peri‐implant disease diagnosis. DL and CNN are the most commonly used tools for the diagnosis of bone levels around teeth or implants, periodontal disease staging and severity, and location of anatomical structures and teeth.

**Conclusion:**

AI and its subsets are promising tools for the diagnosis/risk prediction and treatment planning for periodontal and peri‐implant diseases.

## Graphical abstract

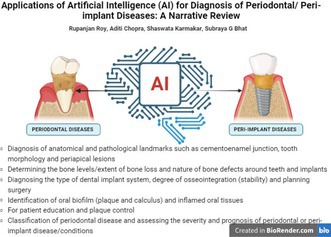

## 1 Introduction

Periodontal disease is a prevalent inflammatory disease affecting nearly 20%–50% of the global population with varying severity [1, 2]. The high prevalence of gingival and periodontal disease is often attributed to poor motivation and oral hygiene maintenance among patients, late diagnosis and ineffective management by clinicians and irregular dental recall visits [3]. Therefore, numerous technological advances and research are being conducted to improve the diagnostic tools for early and more effective diagnosis of periodontal disease.

Recently, AI and various subunits of AI such as deep learning (DL), machine learning (ML), convoluted neural networks (CNNs) and Artificial Neural Network (ANN) are being used to diagnose and plan treatment for periodontal disease more quickly and effectively [4]. AI is being used for patient data segregation, identification of anatomical landmarks, diagnosis and grading of periodontal or peri‐implant disease/conditions. AI is used to assess the clinical features of periodontitis like pocket depth measurement, extent of bone loss, assessing prognosis of periodontal treatment and identification and classification of implant systems [5, 6, 7]. DL, ANNs and CNNs are the most frequently used AI tools for image recognition and classification of periodontal disease using radiographs. AI is being tried for periodontal probing and pocket charting, detecting alveolar bone contour, bone loss, furcation defects, peri‐implant bone levels, occlusal wear, tooth mobility and halitosis. AI also finds its role in implant dentistry for analysing the accuracy of the type of dental implant system, degree of osseointegration and position of the implant placed. Dentists can use patient‐related data along with clinical signs and symptoms of the disease to facilitate faster diagnosis, treatment planning and predict prognosis using DL, CNN and ANN models [4, 7]. With a rapid increase in the application of AI in dentistry, it is crucial to explore current evidence and studies on the role and applications of AI for periodontal and peri‐implant disease diagnosis/treatment plans. Hence, with this review, we aim to discuss and highlight the various evidence and updates on the use of AI for periodontal/peri‐implant diagnosis and treatment planning.

## 2 Search Strategy, Data Screening and Data Extraction Method

A search strategy was developed with the following keywords including Medical Subject Headings (MeSh) terms ((Artificial intelligence [MeSH Terms])) AND (Periodontal disease [MeSH Terms]) and Boolean (AND, OR). The PubMed search string is described as follows: (“periodontal”[Title/Abstract] OR “periodontal disease”[Title/Abstract] OR “periodontitis”[Title/Abstract] OR “periodontal defect”[Title/Abstract] OR “periimplantitis”[Title/Abstract] OR “periimplant disease”[Title/Abstract]) AND (“Artificial intelligence”[Title/Abstract] OR “Machine learning”[Title/Abstract] OR “Deep learning”[Title/Abstract] OR “Convolutional neural network”[Title/Abstract] OR “Artificial Neural network”[Title/Abstract]). An electronic search for English language publications in PubMed, Scopus and Web of Science, was done on 23rd July 2023 and updated on 27 December 2024. Articles in any language were selected. The articles between 2000 to 2024 articles in the last 20 years were selected to provide the most recent evidence. A detailed search string and search strategy and the total number of articles retrieved from each database are mentioned in Table S1. We also checked references of the included studies for other relevant articles. Preprint servers such as Biorxiv, Research Square and IEEE MedRxiv were screened for relevant articles. Conference proceedings and industry studies published in company publications were included. The search results were managed using the Microsoft Office (MS) Excel software (Version 2019).

After the removal of duplicates, two reviewers (R.R. and A.C.) independently selected studies using the inclusion and exclusion criteria: All studies where any AI‐based tools/models were used for diagnosis, or interpretation of radiographs to diagnose any form of periodontal/peri‐implant disease or defects were included. Studies where AI is used for the assessment of defects around implant alone were included. All studies included any form of AI‐based tools or apps for dental radiography to diagnose periodontal/peri‐implant defects. All forms of radiographic interpretation done using grids or manually were included. All observational and clinical studies using human data evaluating the efficacy of AI‐based models compared to conventional methods for the diagnosis of periodontal defects were included. In the first stage, the title and abstract of the studies were independently screened. Studies that did not qualify according to the eligibility criteria and those with inadequate data or information were excluded. This was followed by a full‐text screening of the included studies independently by the four reviewers. Any disagreements regarding inclusion will be resolved initially by discussion between the reviewers (A.C., R.R.). The results of the review are presented as a narrative review synthesis.

## 3 Results

**FIGURE 1:**
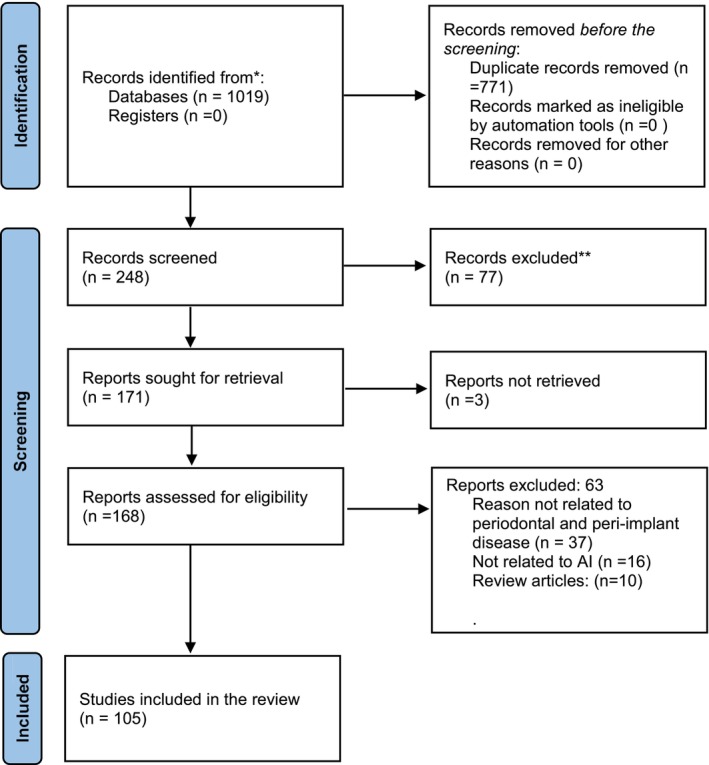
PRISMA Flow diagram.

We retrieved 1019 articles from all databases, of which 248 were included for initial title and abstract screening. During title and abstract screening, 77 articles were excluded and 171 were included in the full‐text screening. During full‐text screening, 105 only were included for the final review (Figure 1). Studies have used various classification algorithms in AI such as ANN, CNN, DL, decision tree (DT) and supporting vector machine (SVM) for periodontal disease diagnosis and classification. Most of the studies have compared the sensitivity and accuracy of AI for periodontal diagnosis to dentists by using previously taken radiographs. The CNN and DL are the most sensitive among all the models used. The detailed characteristics of studies exploring the role of AI in various aspects of periodontal/peri‐implant disease diagnosis are discussed as follows (Table 1).

**TABLE 1:** Studies assessing the role of AI compared convention tools for periodontal/peri‐implant disease diagnosis.

SL/no	Author (year), country, type of study design	Data type	Sample size	Type of AI	Aim/objective of the study	Results & conclusion
**Identification of periodontal disease/condition: Gingival inflammation**						
1.	Al‐Haidan et al. (2014) [8] Cross‐sectional	Patient data	46	Artificial neural networks (ANNs)	Predicted tooth surface loss in individuals using ANNs. Input data consisted of age, smoking status, type of toothbrush, brushing and consumption of pickled food, fizzy drinks, citrus food and dried seeds.	ANN was capable of predicting unknown scores with near 100% degree of accuracy for 2 subjects only out of the 15 (13.3% of subjects). Accepting an error margin of ±5 scores for tooth surface loss results in an accuracy of 73.3%. Modelling tooth surface loss using ANNs is possible and can be achieved with a high degree of accuracy based on patient data
2.	Rana et al. (2017) [9] USA Cross‐sectional	Photographs	405	CNN	Differentiation between inflamed and non‐inflamed tissues to predict gingival disease	The ML classifier provides a pixelwise segmentation of gingival inflammation via intraoral photographs. The ROC curve for classifying inflamed and healthy gingival tissues was found to be 0.746
3.	Feres et al. (2018) [10] Brazil Cross‐sectional	Plaque	435	SVM	To classify patients, utilising machine learning, into generalised chronic periodontitis, generalised aggressive periodontitis and periodontal health using the checkerboard DNA–DNA hybridization method for 40 periodontitis bacteria	The SVM could detect 40 bacterial species and classify species into AGP, CP or healthy. A uniform bacterial composition was seen for healthy samples, unlike the periodontal disease patients, who showed much more diversity and complexity. The relative bacterial load could also help differentiate AgP and ChP
4.	Lee et al. (2018) [11] Korea Cross‐sectional	Periapical radiograph	1740	CNN	To develop a computer‐assisted detection system using deep CNN for the diagnosis and prediction of periodontally compromised teeth	CNN could predict teeth with a hopeless prognosis (ROC curve: 73.4 to 82.6). Deep CNN could predict the prognosis of premolars with an 82.8% accuracy
5.	Yoon et al. (2018) [12] USA Cross‐sectional	Patient dataset	4623	9Weka 3.8 and Deep Neural Network (DNN)‐BigML software for data mining analyses	Development of a prognostic decision support system using patient‐related factors age, acculturation, general health status, soda intake, financial stress, depressive symptom and flossing behaviour	DNN can predict an association between tooth mobility and aging. Soda intake behaviour among age over 55 is a determinant of tooth mobility. An association between a carbohydrate‐heavy diet resulting from agriculture (behaviour) and tooth decay was noted. Younger individuals with a habit of drinking soda and financial stress were more likely to have tooth mobility
6.	Aberin et al. (2019) [13] Philippine Cross‐sectional	Plaque Slides	1000	AlexNet	To determine the efficacy of AlexNet architecture using CNN automated analysis of the microscopic images	CNN could predict gingival health by examining the microscopic images of the dental plaque from slides (75% accuracy)
7.	Askarian et al. (2019) [14] USA Cross‐sectional study	Intraoral photograph	30 [15 diseased and 15 healthy]	SVM	A novel periodontal disease detection method using smartphones, image processing and machine‐learning techniques. A CIELAB colour space was adopted for feature extraction and the support vector machine (SVM) was used for distinguishing healthy and diseased gum	Photographs taken via phones can be used to classify gingival disease via CNN. CNN could detect periodontal infection with 94.3% accuracy, 92.6% sensitivity and 93% specificity, respectively
8.	Moriyama et al. (2019) [15] Japan Cross‐sectional study	Intraoral photograph	820	AlexNet	Development of MapReduce‐like DL model for estimating the pocket depth from oral images.	CNN was able to correlate periodontal disease and intraoral photographs of the diseased area with an accuracy ranging from 78.3% to 84.5%; sensitivity from 50.4% to 74.0% and specificity of around 90%
9.	Alalharith et al. (2020) [16] Saudi Arabia Cross‐sectional study	Intraoral photograph	134	Faster ResNet‐50 CNN	To evaluate object detection and recognition techniques and deep‐learning algorithms for the automatic detection of periodontal disease in orthodontic patients using intraoral images.	Faster R‐CNN can detect teeth (healthy and diseased) with 100% accuracy. It can also detect gingival inflammation with 77.12% accuracy
10.	You et al. (2020) [17] China Cross‐sectional study	Intraoral photograph	886	Deep‐Labv3	Assess the accuracy of a DL model to detect plaque on primary teeth	Compared to the dentist, the AI model demonstrated a higher accuracy (0.736 ± 0.174). The results of a paired t‐test found no significant difference between the AI model and human specialist (*p* > 0.05) in diagnosing dental plaque on primary teeth
11.	Kim et al. (2020) [18] South Korea Case–control study	Saliva	692	SVM	Development of algorithms to predict the severity of periodontitis	CNN can distinguish individuals with healthy and diseased gums and define the severity of periodontal disease by assessing specific microbial and immunological biomarkers
12.	Chen et al. (2021) [19] China Case–control study	Periapical radiograph	2900	CNN	To train CNNs for lesion detections on dental periapical radiographs, to evaluate performances across disease categories and severity levels and train strategies.	CNN could locate sites with periodontal inflammation, decayed teeth and periapical lesions using periapical radiographs. CNNs prefer to detect lesions with severe levels, and it is better to train the CNNs with customised strategies for each disease
13.	Li et al. (2022) [20] China Experimental study	Photographs	3932 oral photos were captured from 625 patients	CNN	To detect gingivitis, calculus and soft deposits from intraoral photographs using a Multi‐Task Learning convolutional neural network (CNN) model	CNN could predict sites with gingivitis and dental calculus. The AUC for detecting gingivitis, calculus and soft deposits was 87.11%; 80.11% and 78.57%, respectively
14.	Shang et al. (2021) [21] China Comparative study	Photographs	7220	U‐Net	Dental calculus, dental caries, tooth wear and gingivitis	U‐Net showed a 10% better image recognition compared to DeepLab‐V3 on oral photographs for assessing tooth wear, carious lesions, dental calculus and gingival inflammation showing that
15.	Shon et al., (2022) [22] Korea Cross‐sectional study	Panoramic OPG	100	U‐Net and YOLOv5	Deep‐learning algorithms to classify and evaluate the periodontal stage. The PBL and CEJ boundaries on the CBNUH image data were detected using U‐Net models. The tooth number and tooth length were identified using the YOLOv5 model	Comparison between the dental specialist and AI showed an n accuracy of 0.929, with a recall and precision of 0.807 and 0.724, respectively all four stages of periodontal disease
**Diagnosis, staging and grading of various periodontal disease**						
16.	Papantonopoulos et al. (2014) [23] Greece Experimental study	Patient data	29	ANN	Development of a model to predict periodontitis based on clinical and immunological markers	ANNs were 90%–98% accurate for periodontitis (AGP or CP) by using immunological cell markers. Datasets of CD4/CD8 cell ratio, levels of CD3 cells, eosinophil, neutrophil, monocyte and lymphocyte counts. The levels of IL‐1, IL‐2, IL‐4, INF‐γ and TNF‐α released from monocytes; and antibody levels against *A. actinomycetemcomitans* and *P.gingivalis* were noted. An ANN obtained the best prediction with cross‐entropy values of monocyte, eosinophil, neutrophil counts and CD4/CD8 ratio as inputs
17.	Bezruk et al. (2017) [24] Ukraine Case–control study	Saliva	141 subjects, 55 healthy and 126 patients diseased.	CNN	Development of a model system to predict periodontitis using the antioxidant level in unstimulated saliva of children and inflammation in periodontal tissues	CNN can predict gingivitis based on levels of salivary lipid peroxidation in the GCF (gingival crevicular fluid). The level of MDA is increased and the level of glutathione is decreased in a group of patients with atopy and it does not depend on the presence of gingivitis in these groups
18.	Yauney et al. (2019) [25] USA Cross‐sectional study	Patient data	1215	EED‐net	An automated process integrates intraoral fluorescent porphyrin biomarker imaging, clinical examinations and a machine‐learning model to investigate the relationship between systemic health conditions and periodontal disease. The modified gingival index and periodontal disease were correlated with a medical history questionnaire, blood pressure, body mass index and imaging of the optic nerve, tympanic membrane and cardiac rhythm	Gingivitis and early periodontal disease were associated with optic nerve abnormalities (*p* < 0.0001) in their retinal scans. We also report significant co‐occurrences of periodontal disease in subjects reporting swollen joints (*p* = 0.0422) and a family history of eye disease (*p* = 0.0337). CNN via using porphyrin biomarkers along with, clinical examinations can predict the systemic health in patients with periodontitis
19.	Chang et al. (2020) [20] South Korea Cross‐sectional study	Panoramic OPG	340	ResNet	A deep‐learning architecture to detect radiographic bone loss to classify periodontal diseases	DL architecture and the conventional CAD approach demonstrated high accuracy and excellent reliability in the automatic diagnosis of periodontal bone loss and staging of periodontitis. The Pearson correlation coefficient of the automatic method with the diagnoses by radiologists was 0.73 overall for the whole jaw (*p* < 0.01), and the intraclass correlation value was 0.91 overall for the whole jaw (*p* < 0.01)
20.	Chen et al. (2020) [26] China & UK Cross‐sectional study	Photographs	180	ANN	Development of a gingivitis recognition program based on Grey‐level co‐occurrence matrix, ANN and genetic algorithm	ANN showed 71% to 75.44% accuracy in detecting gingivitis from photographs
21.	Farhadian et al. (2020) [27] Iran Cross‐sectional study	Patient data	320	SVM	Designing and developing SVM model to diagnose periodontal diseases	Support Vector Machine (SVM) effectively diagnoses various periodontal diseases. The model using the radial kernel function achieved an overall classification accuracy of 88.7% through tenfold cross‐validation. Specific accuracies included 96.0% for gingivitis, 64.0% for localised periodontitis and 92.2% for generalised periodontitis. An evaluation using the HUM criteria resulted in an overall HUM value of 0.912
22.	Thanathornwong et al. (2020) [28] Thailand Cross‐sectional study	Panoramic OPG	100	Faster R‐CNN model ResNet architecture	Proposed a deep learning‐based object detection method to identify periodontally compromised teeth on digital OPG.	Faster R‐CNN trained on a limited amount of labelled imaging data performed satisfactorily in detecting periodontally compromised teeth. The mean precision and recall rate were 0.81 and 0.80. The sensitivity and specificity of 0.84 and 0.88 were reported with an F‐value of 0.81
23.	Khaleel et al. (2021) [29] Iraq Comparative study	Photographs	120	The BAT swarm algorithm, the Self‐Organising Map (SOM) algorithm and the Fuzzy Self‐Organising Map (FSOM)network algorithm were used to diagnose Gingivitis disease	To assess the accuracy of algorithm models to diagnose gingival diseases	BAT showed the highest accuracy for diagnosis of Gingivitis disease equal (97.942%)
24.	Kabir et al. (2021) [30] USA Cross‐sectional study	Periapical radiograph	700	CNN combined with ResNet and U‐Net	Development of DL network HYNETS (Hybrid network for periodontitis stages from radiographs) for classifying patients with periodontitis	HYNETS achieved the average dice coefficient of 0.96 and 0.94 for the bone area and tooth segmentation and the average AUC of 0.97 for periodontitis stage assignment. CNN combined with OPGs can be used for staging and grading periodontal disease
25.	Li et al. (2021) [31] China Comparative study	Photographs	110 images	CNN [DeepLabv3+ network with Xception and MobileNetV2]	Detection of gingivitis, dental calculus and soft deposits using multi‐tasking learning CNN model	The proposed network model could predict the contour of the site‐specific gingival area and accurately categorise gingival inflammation. The mean intersection‐over union (mIOU) with Xception65 as the backbone network was 0.379 and 0.485, respectively, and the mIOU with MobileNetV2 as the backbone network was 0.355 and 0.449, respectively
26.	Ossowska et al. (2022) [32] Japan Retrospective study	Clinical parameters	110	CNN	Assessed the efficacy of ANN to detect the grade and severity of periodontal disease. The study used patient data (demographic and clinical parameters such as age, gender, use of smoking); plaque scores, bleeding on probing, pocket depth and clinical attachment loss	No statistically significant differences in the clinical periodontal assessment with ANN. A sensitivity of 85.7% and specificity of 80.0% were noted for the diagnosis of periodontal disease. The incorrectly classified patients according to the grade of periodontitis were 15.8%
27.	Yang et al. (2023) [33] Animal study	CBCT images	108 Yorkshire pig mandibles	3D soft tissue reconstruction	To compare the virtual and clinical measurements to predict gingival phenotype	The clinical and virtual measurements were positively correlated (*r* = 0.9656, *p* < 0.0001). No difference in the agreement between the virtual and clinical measurements for the pre‐pubertal samples (0.033 ± 0.195 mm) and matured samples (0.087 ± 0.240 mm) were noted. Noticeably, there is a greater agreement between the virtual and clinical measurements at the buccal sites (0.019 ± 0.233 mm) than at the lingual sites (0.116 ± 0.215 mm) No statistical difference in reproducing the data between the virtual and clinical methods were noted
28.	Patel et al. (2022) [34] USA Experimental study	Electronic Data Record (EDR)	27 138	XGBoost ML model	Developing Automated Computer Algorithms to Phenotype Periodontal Disease Diagnoses in Electronic Dental Records	The completeness of periodontal disease diagnosis from the record showed that ML can be effectively used. The periodontal diagnosis codes were 36%; diagnoses in clinical notes was 18%, and for charting information was 80%. Eleven percent of patients had healthy periodontium, 43% were with gingivitis, 3% with stage I, 36% with stage II and 7% with stage III/IV periodontitis
**Identification of periodontal disease or condition, prognosis, including degree of alveolar bone loss**						
29.	Kim et al. (2022) [35] South Korea Cross‐sectional study	Panoramic	12 179	DeNTNet	Presence or absence of bone for each tooth	CNN was effective in automatically detecting bone loss. Compared to the dentists, the F1 score of the DeNTNet was 0.75, which was higher compared to dentists (0.69)
30.	Krois et al. (2019) [36] Germany Cross‐sectional study	Panoramic OPG	85	CNN	Bone loss (%) was calculated for each tooth manually by determining the distance between the CEJ and the alveolar crest divided by the CEJ's distance to the tooth's apex.	The classification accuracy for the CNN was 0.81 ± 0.02. The mean sensitivity and specificity for CNN were 0.81 ± 0.04 and 0.81 ± 0.05, respectively. The accuracy of the dentists was 0.76 ± 0.06. The CNN was not statistically significantly superior to the examiners (*p* = 0.067/t‐test). The mean sensitivity and specificity of the dentists were 0.92 ± 0.02 and 0.63 ± 0.14, respectively
31.	Huang et al. (2020) [37] China Case–control study	Gingival crevicular fluid	25	SVM	To compare the levels of 20 proteins related to periodontal disease in the gingival crevicular fluid between healthy and periodontitis patients using five periodontitis five classification models	Seven proteins such as matrix metalloproteinase, Interleukin [(L)‐1α, IL‐1β, IL‐8, osteoactivin, osteoprotegerin and C‐reactive protein] were significantly upregulated in patients with periodontitis compared to healthy controls. The highest diagnostic accuracy using a ROC curve was observed for IL‐1β. Linear discriminant analysis had the highest classification accuracy across the five tested classification models.
32.	Li et al. (2020) [38] China Cross‐sectional study	Panoramic OPG	302	R‐CNN	Propose an interpretable method called Deetal‐Perio to predict the severity degree of periodontitis in dental panoramic radiographs. Deetal‐Perio segments the contour of the alveolar bone and calculates a ratio for the individual tooth to represent bone loss	Deetal‐Perio predicts the severity degree of periodontitis given the teeth' ratios. CNN models for bone loss recognition and found R‐CNN to be the most effective
33.	Moran et al. (2020) [39] Brazil Cross‐sectional study	Periapical radiograph	467	ResNet, Inception	Classified the regions using radiographs according to the presence of periodontal bone destruction.	Accuracy, precision, recall and specificity were found to be 0.817, 0.762 and 0.923
34.	Shimpi et al. (2020) [40] USA Cross‐sectional study	Patient data	N/A	Naïve Bayes (NB), Logistic Regression (LR), SVM, ANN, DT.	Tested the new PD risk assessment model using supervised machine learning methods.	ANN and DT were found to be more accurate for classifying the risk of periodontitis compared to LR NB and SVM. DT model showed a sensitivity and specificity of 87.08% and 93.5%
35.	Moran et al. (2021) [41] Brazil Cross‐sectional study	Periapical radiograph	5	CNN models	To perform a qualitative and quantitative analysis of how different super‐resolution algorithms (nearest, bilinear, bicubic, Lanczos, SRCNN and SRGAN) used on periapical images can impact the assessment of periodontal bone loss	DL methods, especially SRGAN were able to improve the resolution of the images and provided better visualisation the bone loss. However, the DL model was not found to be effective in improving the classification accuracy of CNN as it was found to increase the occurrence of minor artefacts that influence the texture and patterns of the image
36.	Danks et al. (2021) [42] United Kingdom Cross‐sectional study	Periapical radiograph	340	ResNet	A deep neural network with hourglass architecture to predict dental landmarks in single, double and triple‐rooted teeth using periapical radiographs	The accuracy of landmark localization achieved percentage Correct Key points (PCK) of 88.9%, 73.9% and 74.4%, respectively, and a combined PCK of 83.3% across all root morphologies, outperforming the next best architecture by 1.7%. When compared to clinicians' visual evaluations of full radiographs, the average PBL error was 10.69%, with a severity stage accuracy of 58%. This simulates current interobserver variation, implying that diverse data could improve accuracy
37.	Lee et al. (2022) [43] South Korea Cross‐sectional study	Panoramic OPG	530	U‐Net, Dense U‐Net, ResNet, SegNet	A deep‐learning model was developed by integrating three segmentation networks (bone area, tooth, cementoenamel junction) and image analysis to measure the bone level and measure bone loss stages. RBL percentage, staging and presumptive diagnosis were compared with the measurements and diagnoses made by the independent examiners.	No significant difference was noted for the level of bone loss measured by the DL and examiners (*p* = 0.65). The area under the receiver operating characteristics curve of RBL stage assignment for stages I, II and III was 0.89, 0.90 and 0.90, respectively. The accuracy of the case diagnosis was 0.85 DL model was found to be effective in measuring the RBL measurements and image‐based periodontal diagnosis using periapical radiographic images. The accuracy of the model to detect bone loss was found to be 79.54%
38.	Tsoromokos et al. (2022) [44] Netherlands Cross‐sectional study	Mandibular periapical radiographs	1546 approximal sites were manually automated [training set (*n* = 1308 sites), a validation set (*n* = 98 sites) and a test set (*n* = 140 sites)]	13‐layered CNN	To develop a CNN algorithm to recognise the cementoenamel junction, the most apical extent of the alveolar crest, the apex and the surrounding alveolar bone.	Percentage of alveolar bone loss and angular defects. The CNN‐trained algorithm on radiographs showed a diagnostic performance with moderate to good reliability in detecting periodontal bone loss. The mean value for %ABL based on MA was 25.7% ±12.3% for non‐molars and the corresponding mean value for the CNN %ABL was 22.3% ± 11.3%. The mean value for %ABL based on MA was 50.6% ± 8.3% for sites with ABL ≥ 33%, and the corresponding mean value for the CNN %ABL was 46.0% ± 11.6% (ICC value of 0.641; 95% CI, 0.227–0.855; *p* < 0.05). The mean difference between MA and CNN was 4.5% ± 7.9%
39.	Kurt‐Bayrakdar et al. (2024) [45] Turkey Cross‐sectional study	Panoramic OPG	1121	CNN: U‐Net architecture	To develop and assess the efficacy of a CNN‐based AI system to detect periodontal bone loss and patterns of bone destruction	Highest in the detection of total alveolar bone loss; lowest: Vertical bone loss. CNN algorithms provide more detailed information such as automatic determination of periodontal disease severity and treatment planning in various dental radiographs
40.	Zadrozny et al. (2022) [46] Poland Cross‐sectional study	Panoramic OPG	100	Diagnocat	Assessed the reliability of the AI automatic evaluation of the marginal and apical periodontium.	AI protocol showed very high specificity (above 0.9) in all assessments compared to ground truth except for periodontal bone loss. The reliability for caries assessment (ICC = 0.681) and periapical lesions assessment (ICC = 0.619) was unacceptable. The time required for diagnosis by dentists and AI was 8.5 min and 2 min, respectively
41.	Jiang et al., (2022) [47] China Cross‐sectional study	Panoramic OPG	640	DL: U‐Net and YOLO‐v4	Localise the tooth and key points (mesial CEJ, distal CEJ, root apex and the deepest alveolar crest on the mesial and distal side) for measuring periodontal bone loss.	Percentage of periodontal bone loss; vertical and furcation lesions recognition were detected. CNN was able to diagnose the different stages of periodontal disease. In stage I lesions, the sensitivity of the model was 0.76; while it was 0.57 for dentists. For stage II lesions, the sensitivity of the model was 0.75, while it was 0.46 for dentists. For stage III lesions, the sensitivity of the model was 0.81, while it was slightly higher at 0.82 for dentists. It is feasible to establish a deep deep‐learning model for the assessment of bone loss
42.	Sunnetci et al. (2022) [48] Turkey Cross‐sectional Study	Panoramic radiographs	1432 images	AlexNet‐based deep and SqueezeNet‐based deep image features.	To discriminate the presence and absence of periodontal bone loss using AlexNet and SqueezeNet architectures.	Among the AlexNet‐based classifiers, Linear SVM was best with an accuracy of 81.49%. Among the SqueezeNet‐based classifiers, the Medium Gaussian SVM showed the best results. Linear SVM classifier is better than other classifiers in terms of accuracy, error, sensitivity and F1 score values. The error, sensitivity, specificity, precision and F1 score values of this classifier are equal to 18.51%, 84.57%, 79.14%, 75.68% and 79.88, respectively
43.	Widyaningrum et al., (2022) [49] Indonesia Cross‐sectional study	Digital panoramic radiographs.	100	Multi‐Label U‐sNet and Mask R‐CNN models	Assessed the ability of CNN to perform image segmentation for periodontitis staging. Normal conditions and 4 stages of periodontitis were annotated on these panoramic radiographs. The performance of the segmentation models against the radiographic diagnosis of periodontitis conducted by a dentist was described by evaluation metrics	Multi‐Label U‐Net produced superior image segmentation to that of Mask R‐CNN. Multi‐Label U‐Net achieved a dice coefficient of 0.96 and an IoU score of 0.97. Meanwhile, Mask R‐CNN attained a dice coefficient of 0.87 and an IoU score of 0.74. U‐Net showed the characteristic of semantic segmentation and Mask R‐CNN performed instance segmentation with accuracy, precision, recall and F1‐score values of 95%, 85.6%, 88.2% and 86.6%, respectively
44.	Hoss et al., (2023) [50] Germany Cross‐sectional study	Periapical radiographs	21 819	ResNet‐18 (ACC), MobileNetV2, ConvNeXT/s, ConvNeXT/b and ConvNeXT/l	Compared the data of different types of CNN networks for the detection of PBL	All five CNNs, ResNet‐18 (ACC 82.8%; AUC 0.884), MobileNetV2 (82.0%; 0.884), ConvNeXT/s (83.9%; 0.903), ConvNeXT/b (84.8%; 0.911) and ConvNeXT/l (84.2%; 0.913) showed the same performance. The CNNs achieved an overall ACC between 82.0% and 4.8%. When considering the ability of the tested CNNs to detect PBL with sextants on periapical radiographs, differences between teeth in the lower and upper jaw were observed
45.	Vollmer et al. (2023) [51] Germany Cross‐sectional study	Panoramic OPG	1414	Keypoint R‐CNN with a ResNet‐50‐FPN	To develop a multi‐object detection algorithm to detect and quantify radiographic bone loss using standard two‐dimensional radiographic images in the maxillary posterior region.	The average precision and recall over all five folds were 0.694 and 0.611, respectively. Mean average precision and recall for the key point detection were 0.632 and 0.579, respectively
**Identification of dental implant and peri‐implant disease/conditions**						
46.	Takahashi et al. (2020) [52] Japan Cross‐sectional study	Panoramic OPG	1282	YOLO v3 with fine‐tuning with TensorFlow and Keras deep‐learning libraries	To identify dental implant systems using a DL method. An object detection. The true positive ratio and average precision of each implant system were noted	Dental implants could be identified using deep learning‐based object detection via OPGs. True positive and mean precision for each implant system ranged from 0.50 to 0.82 and from 0.51 to 0.85, respectively
47.	Papantonopoulos et al. (2017) [53] Greece Cross‐sectional study	Patient data and OPG	72 implant‐treated patients with 237 implants	k‐means method guided by multidimensional unfolding for detecting individual implant mean bone levels. Principal component analysis (PCA) as a variable reduction method for an ensemble selection (ES) and a support vector machines models (SVMs).	To identify possible implant ‘phenotypes’ and predictors of individual implant mean bone levels	Two implant ‘phenotypes’ were identified, one with susceptibility and another with resistance to peri‐implantitis. Prediction of IIMBL could be achieved by using six variables such as number of teeth, full‐mouth plaque scores, implant surface, periodontitis severity, age and diabetes. The cluster susceptible to peri‐implantitis showed a mean individual implant mean bone levels of 5.2 mm and included implants placed mainly in the lower front jaw and in mouths having a mean of eight teeth. ES and SVMs showed Network analysis revealed limited interdependencies of variables among peri‐implantitis‐affected and non‐affected implants
48.	Lee et al. (2020) [54] South Korea Cross‐sectional study	5390 panoramic and 5380 periapical radiographs	10 770	GoogLeNet Inception v3	To evaluate the efficacy of the deep CNN algorithm for the identification and classification of 3 types of dental implant systems. Performed image Assessed the accuracy, sensitivity, specificity, receiver operating characteristic curve, area under the receiver operating characteristic curve (AUC) and confusion matrix compared between deep CNN and periodontal specialist	Deep CNN architecture showed an AUC of 0.971 and the board‐certified periodontist showed an AUC of 0.925. CNN architecture was useful for the identification and classification of dental implant systems
49.	Sukegawa et al. (2020) [55] Japan Cross‐sectional study	Panoramic OPG	8859	CNN (finely tuned VGG‐16; VGG1‐9 and normal transfer‐learning VGG‐16)	To classify and clarify the accuracy of different dental implant brands via five deep CNN models (specifically, a basic CNN with three convolutional layers, VGG16 and VGG19 transfer‐learning models and finely tuned VGG16 and VGG19).	The finely tuned VGG1‐6 CNN model, followed by transfer‐learning VGG‐16, showed the highest accuracy for classifying dental implants. The finely tuned VGG19 was second best, followed by the normal transfer‐learning VGG16. We confirmed that the finely tuned VGG16 and VGG19 CNNs could accurately classify dental implant systems from 11 types of implant systems
50.	Cetiner et al. (2021) [56] Turkey Cross‐sectional study	Patient data	216 patients with 542 dental implants	MLP, DT, ANN	To determine a predictive decision model for peri‐implant health and disease.	The DT model was the most accurate (0.871) followed by ANN (0.852) and Logistic regression (0.832)
51.	Lee et al. (2021) [57] South Korea Cross‐sectional study	Panoramic and Periapical radiographic images.	21 398 dental implants (251 intact and 194 fractured DI radiographic images)	Deep CNN; architectures (VGGNet‐19, GoogLeNet Inception‐v3) and automated Deep CNN.	To evaluate the reliability and validity of three different deep convolutional neural networks (DCNN) architectures (VGGNet‐19, GoogLeNet Inception‐v3 and automated DCNN) for the detection and classification of fractured dental implants	Deep CNN architectures showed an accuracy of over 0.80 AUC for fractured dental implant detection and classification. DCNN architecture showed the highest and most reliable results for detection (AUC = 0.984) and classification (AUC = 0.869) compared to fine‐tuned and pre‐trained VGGNet‐19 and GoogLeNet Inception‐v3 architectures
52.	Mameno et al. (2021) [56] Japan Retrospective cohort study	Patient data	254 implants, 127 with and 127 without peri‐implantitis, from among 1408 implants	Logistic regression, support vector machines and random forests (RF).	To create a model for predicting the onset of peri‐implantitis using ML methods. Demographic data and risk factors for the development of peri‐implantitis were analysed	RF had the highest performance in predicting the onset of peri‐implantitis (AUC: 0.71, accuracy: 0.70, precision: 0.72, recall: 0.66 and f1‐score: 0.69). The factor that influenced prediction most was implant functional time, followed by oral hygiene. Use of 50% to 60%, smoking more than 3 cigarettes/day, keratinized mucosa width of less than 2 mm, and the presence of less than two occlusal supports tended to be associated with an increased risk of peri‐implantitis. Peri‐implantitis onset was predicted in 70% of cases by RF, which allows consideration of nonlinear relational data with complex interactions
53.	Ning et al. (2021) [58] Germany Case–control study	Saliva	Two periodontitis‐related GEO transcriptomic datasets (GSE16134 and GSE10334)	DisGeNet, HisgAtlas	This study applied a DL‐based AE for the first time to identify immune subtypes of periodontitis and pivotal immunosuppression genes (Differentially expressed genes (DEGs), signalling pathways and transcription factors (TFs)) that differentiate periodontitis and healthy patients	Three ‘master’ immunosuppression genes, PECAM1, FCGR3A and FOS were identified as candidates pivotal to immunosuppressive mechanisms in periodontitis. Six transcription factors, NFKB1, FOS, JUN, HIF1A, STAT5B and STAT4, were identified as central to the TFs‐DEGs interaction network. Key signalling pathways and TF‐target DEGs that putatively mediate immune suppression in periodontitis were identified. PECAM1, FCGR3A and FOS emerged as high‐value biomarkers and candidate therapeutic targets for periodontitis. DL can predict the genes linked with immune suppression during periodontitis (accuracy: 92.78%).
54.	Wang et al. (2021) [59] USA Cohort study	Peri‐implant granulation tissue	N/A	FARDEEP	CNN can be used as a processing tool for identifying and differentiating patients with peri‐implantitis based on the clinical, immunological and microbial profile.	Low‐risk patients exhibited elevated M1/M2‐like macrophage ratios and lower B‐cell infiltration. The low‐risk immune profile was characterised by enhanced complement signalling and higher levels of Th1 and Th17 cytokines. *Fusobacterium nucleatum* and *Prevotella intermedia* were significantly enriched in high‐risk individuals
55.	Liu et al., (2022) [60] China Cross‐sectional study	Periapical radiograph	1670 periapical radiographic images (*n* = 1370), validation (*n* = 150) and test (*n* = 150) datasets.	R‐CNN	The accuracy of an artificial intelligence (AI) application for the detection of marginal bone loss on periapical radiographs.	AI was comparable to the dentist in detecting bone loss. The agreement between the AI system and expert was moderate to substantial (κ = 0.547 and 0.568 for bone loss sites and bone loss implants, respectively) for detecting marginal bone loss around dental implants. AI system based on Faster R‐CNN analysis of periapical radiographs is a promising tool for peri‐implant marginal bone level detection
56.	Gardiyanoğlu et al., (2023) [61] Cyprus Cross‐sectional study	Panoramic OPG	8138 OPGs	Computer Vision Annotation Tool (CVAT)	To provide automatic segmentation of various objects (teeth, crown–bridge restorations, dental implants, composite‐amalgam fillings, dental caries, residual roots and root canal fillings) on the OPGs	The diagnosis made by the CVAT and the dentist was positively correlated (0.99) with a reliability of 0.989 (*p* = 0.947)
57.	Rekawek et al. (2023) [62]. United States Retrospective study	Patient data	398 unique patients receiving a total of 942 dental implants	Logistic regression, random forest classifiers, support vector machines	To develop an ML model to predict dental implant failure and peri‐implantitis	The random forest model gave the maximum predictive performance (ROC = 0.872 and AUC = 0.840 for dental implant failures and peri‐implantitis, respectively). Factors such as the amount of local anaesthesia, length and diameter of implant, use of antibiotics preoperatively and frequency of dental visits for oral prophylaxis were found to be associated with implant failure. Factors associated with peri‐implantitis were implant length, implant diameter, use of preoperative antibiotics, frequency of hygiene visits and presence of diabetes mellitus
58.	Oh et al. (2023) [63] South Korea Cross‐sectional study	Panoramic and periapical radiographs	580 patients (1206 dental implants) divided into two groups radiographed immediately after implant placement and Group 2 with implants radiographed after confirming successful osseointegration	Seven deep‐learning models: ResNet‐18,34,50; DenseNet‐121 201; MobileNet‐V2 and MobileNet‐V3	To predict the degree of osseointegration in dental implants using DL.	Osseointegration of dental implants can be predicted to some extent through deep learning using plain radiography. The mean specificity, sensitivity and accuracy of the deep‐learning models were 0.780–0.857, 0.811–0.833 and 0.799–0.836, respectively. Furthermore, the mean AUROC values ranged from 0.890–0.922. The best model yields an accuracy of 0.896 and the worst model yields an accuracy of 0.702
59.	Vera et al. (2023) [64] Spain Cross‐sectional	Periapical and bitewing radiographs	2920 periapical/bitewing radiographs	DL‐object‐detector (YOLOv3)	To develop automatic artificial intelligence techniques for determining bone loss. The tool aimed to identify prosthesis (crown), implant (screw), screw edges and identify significant points (intensity bone changes, intersections between screw and crown). Distances between these points are used to compute bone loss	The image understanding performance was found to be satisfactory. The statistical average/standard deviation in pixels for line fitting (2.75 ± 1.01) and for significant point detection (2.63 ± 1.28) was according to the dentist opinions

Abbreviations: AGP, Aggressive Periodontitis; ANN, Artificial neural network; CNN, Convoluted Neural Network; CP, Chronic Periodontitis; DNN, Deep neural network; EED, Enhanced Encoder‐Decoder Network; MLP, multilayer perceptron; OPGs, Orthopantomogram; R‐CNN, Regional convoluted neural network; RecAUC, area under the curve; ResNet, Residual Network; ROC, receiver operator characteristic (ROC) curves; SVM, Support vector machine; U‐Net, U‐shaped encoder‐decoder; Yolo v4, You only look once version 4.

### 3.1 Evidence on the Use of AI for the Diagnosis and Classification of Periodontal Disease

CNNs can process high‐resolution 2D or 3D radiographs to locate, classify and grade various periodontal diseases. AI‐based models are used to identify anatomical landmarks such as the number of decayed, missing and filled teeth; nature of prosthesis (crowns, bridges, implant); location of cementoenamel junction; morphology of the crown and root of the teeth; location of cementoenamel junction (CEJ); locate periodontally compromised/mobile teeth; determine the extent of tooth resorption and alveolar bone loss; presence of a periapical radiolucency and root fractures [7, 65, 66]. ANN models are also used to classify oral malodor by using the microbial profile of saliva and assessing the periodontal pathogens. Various metabolites and biomarker analyses using saliva, plaque, gingival crevicular fluid (GCF) and serum are being done using AI for predicting the nature of various oral and periodontal diseases such as gingivitis, cancer, aphthous ulcers, etc. (Table 1) [6]. Ozden et al. compared the efficacy of SVM, DT, ANN and CNNs and found that SVM, DT and ANNs can best classify periodontal disease based on signs and symptoms. However, DT and SVM have the highest degree of accuracy and ANN showed the worst relation, with an accuracy of only 46% [67]. Tabatabaei et al. also used CNN for the diagnosis of gingival and periodontal disease using intraoral images and found that the CNN networks have an accuracy of only 66.7% compared to conventional techniques. However, the accuracy can be improved if a correlation of radiographs is made with the case history of the patient and associated clinical symptoms like Bleeding on Probing (BOP), clinical attachment loss (CAL), probing depth (PD), mobility and status of the pulp (vitality). Shankarapillai et al. found that forward ANNs with propagation algorithm of Levenberg‐Marquardt training function can effectively diagnose periodontal disease using the PD, bone levels and immunologic parameters and can even differentiate different forms of gingivitis and periodontitis [68]. Arbabi et al. also noted that by including demographic parameters like age and gender along with clinical parameters such as plaque index, PD and CAL with ANN and LM algorithms, the output and detection rate can be improved [69].

Nagane et al. and Mago et al. also tried to use patient‐related data and ML models to predict periodontally compromised teeth [70, 71]. Nagane et al. used the ML system to predict periodontal diseases based on age, gender, signs and symptoms, nature of risk factors such as genetics, obesity, stress, medications and type of image. The authors compared the diagnostic accuracy of AI to identify and predict the probability of gum diseases with that of a standard dentist by using Dumpster‐Shaffer Reasoning and found this system to be promising for developing mobile apps for personalised detection of gum disease [71]. Mago et al. predicted the effect of periodontal treatment for mobile teeth by assessing the pain scores, degree of infection and tooth mobility and occlusal trauma by using a fuzzy expert system (FES). The authors also assessed seven treatment options for mobility splinting, use of Fixed Partial Denture (FPD), extraction, scaling and root planing, occlusal corrections, use for medications and endodontic treatment. The number of treatment options provided by the system and the dentist was compared using the Chi‐square test of significance. The study found that dentists tend to give more treatments for mobile teeth (mainly advice medications and extraction) [72]. Thanathornwong et al. also used a DL to identify compromised/hopeless teeth via digital OPGs. The identification of periodontally compromised teeth by a periodontist was considered the ground truth. The study found that the R‐CNN has a precision rate of 0.81. CNN excluded the healthiest teeth areas with good scores for both sensitivity (0.84) and specificity (0.88) [28]. Allahverdi and Akcan also used FES to diagnose periodontal disease by obtaining inputs from clinical signs and symptoms such as gingival index, PD, CAL, tooth mobility, extent of bone loss and risk factors data and found that FES can diagnose the severity and stage of the gingival disease and provide treatment options. This can help dentists by reducing the time taken for data collection and for the manual method of decision‐making [73]. Yoon et al. applied the DL algorithms to predict tooth mobility in Latinos and found that the age of the patient, medical condition, the amount of consumption of acidic drinks (soda), use of floss and financial stress are some of the strongest parameters that correlate with the self‐reported tooth mobility.

AI‐based model (DL/Bat heuristic optimization algorithm/CNN/DNN) are also been used to identify the inflamed disease sites by assessing the changes in the pixel level from intraoral photographs (Table 1) [9, 12, 13, 17, 19, 24, 26, 29, 31]. Several studies have used AI models to detect gingival inflammation from intraoral photographs with good accuracy (0.47 to 0.83) [18, 23] (Table 1) [6]. The AI system was able to clinically predict gingivitis and detect non‐inflamed (healthy) sites [53]. Li et al. used intraoral photographs from 110 patients trained DL software to detect inflamed and non‐inflamed sites and classified the patients into four health statuses (healthy, questionable healthy, questionable diseased and diseased). The status diagnosis with the DL model (Deep‐Labv3 network along X‐caption and MobileNet‐V2) was confirmed and compared with the dental specialist with more than 15 years of clinical experience. The authors found that the DL approach was as effective in diagnosing the diseased condition as the dentist and this system can be used for dental self‐check‐ups by using the mobile app [31]. Khaleel et al. tested the efficacy of the Self‐Organising Map (SOM) and Bat swarm algorithm' for the diagnosis of gingival diseases/conditions such as abscess, oral lichen planus, gingival enlargement, gingival melanoma or pigmentation, gingival recession, oral ulcer. Among all these methods, the testing state of the Bat swarm algorithm showed a higher accuracy for the diagnosis of gingivitis (97.942%) [29]. You et al. also used the DL model to detect plaque in children by using intraoral photographs of primary dentition and found that the AI model can detect dental plaque more efficiently compared with an experienced paediatric dentist [9]. CNN model (AlexNet) can assess microscopic dental plaque images for predicting the status of the periodontium (health or periodontitis) by using slides made from dental plaque [17]. Chen et al. developed a gingivitis recognition program based on ANN, Grey‐level co‐occurrence matrix and genetic algorithm and found that ANN had a 71% to 75.44% accuracy in detecting gingivitis from photographs.

Various metabolites in biological fluids such as saliva, gingival crevicular fluid (GCF), serum and dental plaque samples are being tested using AI‐based model (ANN/CNN) to diagnose periodontal disease. For example, Bezrukzk et al. used CNN to detect salivary lipid peroxidation for the diagnosis of periodontal disease [24]. ANN models are also used to classify patients with aggressive or chronic periodontitis based on the levels of immunologic parameters (levels of immune cells such as CD4, CD8, CD3, monocytes, eosinophils, neutrophils and lymphocytes; levels of inflammatory markers such as TNF, IL‐1/2/4, INF‐γ, antibody levels against periodontal pathogens such as *A. actinomycetemcomitans* and *P. gingivalis* . Studies have shown that using these parameters, the ANN model can achieve 90%–98% accuracy in classifying periodontitis patients. The overall prediction was best given by an ANN with CE of monocyte, eosinophil, neutrophil counts and CD4/CD8 ratio as inputs [23, 53]. Kim et al. assessed the efficacy of ML models in predicting the severity of chronic periodontitis by analysing the microbial bacteria ( *Tannerella forsythia* , *Porphyromonas gingivalis* , *Campylobacter rectus* , *Fusobacterium nucleatum* , *Aggregatibacter actinomycetemcomitans* , *Treponema denticola* , *Prevotella intermedia* , *Peptostreptococcus anaerobius* and *Eikenella corrodens* ). Mouthwash samples were collected from healthy and patients with chronic periodontitis. The microbial profile and severity of periodontal destruction were assessed. The accuracy of the ML model was found to be highest for models that were classified as ‘healthy’ and with ‘moderate or severe’ periodontitis. The levels of *Tannerella forsythia* , *Porphyromonas gingivalis* and *Treponema denticola* were found to be positive indicators of distinguishing chronic periodontitis from healthy controls [18]. Feres et al. also used bacterial species to classify patients into healthy and periodontitis (chronic and aggressive periodontitis) by utilising ML models by checking the composition of biofilm in healthy and diseased patients. The results showed a uniform bacterial composition in healthy patients, whereas the bacterial composition of periodontally diseased samples was more diverse. These changes in the relative bacterial load were used to develop an ML model that can distinguish between the chronic and aggressive nature of periodontitis [10]. Huang et al. assessed the levels of twenty biomarkers for periodontal disease in GCF in healthy individuals and individuals with severe periodontitis. The levels of the biomarkers in both healthy and diseased individuals were used to build receiver operator characteristic (ROC) curves and compare five classification models, including Linear discriminant analysis and classification model, Regression Trees, SVM, k‐nearest neighbour and random forest. Seven markers (Interleukins [IL]‐1α, IL‐1β, IL‐8, matrix metalloproteinase‐13, osteoactivin, osteoprotegerin and C‐reactive protein) were significantly upregulated in periodontitis patients as compared to healthy controls. The accuracy to diagnose was highest for IL‐1β (AUC: 0.984). Linear discriminant analysis showed the maximum accuracy in classifying patients with periodontitis [37].

SVM models are also being used to grade and stage periodontal diseases. Moran et al. compared the efficacy of the ‘ResNet’ and ‘Inception model’ for the diagnosis of periodontal disease. The authors found that the Inception model was a better diagnosis even with a small data set [41]. In 2021, the authors again compared the effects of different image improvement methods using DL systems like bilinear, nearest, Lanczos, SRCNN, bicubic and SRGAN. DL was found to improve the visual quality of the intraoral images but was found to be less useful for disease classification [39]. Farhadian et al. collected from 300 periodontitis patients and used 11 variables (gender; age; smoking status; gingival, plaque, bleeding index, mobility and oral hygiene index; PD; percentage alveolar bone loss based on radiographs) for assessing the periodontal status. These variables were to classify the periodontal disease status as gingivitis, localised periodontitis or generalised periodontitis. The study found that the SVM model could detect the mean difference in CAL, plaque index and PD among patients with gingivitis, localised periodontitis and generalised periodontitis effectively with an overall classification accuracy of 88.7% [27]. Li et al. using a new AI‐based method could diagnose chronic gingivitis based on the 'Particle Swarm Optimization Neural Network' (PSONN)' and ‘Multi‐Channel Grey Levels Occurrence Matrix’. The model was trained to differentiate between different shades of grey by using inputs from intraoral photographs of healthy and inflamed gingival tissues [74]. Chau et al. used Deep‐Labv‐3 built on Keras with TensorFlow 2 to diagnose gingivitis using intraoral photographs. The gingival conditions of individual sites were labelled as healthy, diseased or questionable. AI could correctly diagnose 11.14 lakhs of healthy and 11.83 lakhs of diseased pixels with sensitivity (0.92) and specificity (0.94), which is comparable to the manual examination by human dentists [75]. Alalharith et al. also used intraoral images to locate teeth and detect gingival inflammation by using ResNet‐50 Faster Region‐based CNN and found 100% detection accuracy and precision [16]. Some authors have explored the use of AI‐based diagnosis using photographs to assess the degree of plaque control and home oral hygiene routine of the patient [14, 15] (Table 1). Some studies have tried to grade periodontal disease based on the new classification system of periodontal and peri‐implant diseases and conditions using dental panoramic radiographs have also been developed. A new hybrid framework of DL to detect periodontal bone loss on teeth by using CEJ as the reference point on the radiographs was developed. The DL model could detect periodontal disease more efficiently compared to a dentist. The diagnosis by the radiology specialist was 0.73 and the comparison between DL and the radiologist should have a correlation value of 0.91 (*p* < 0.01) [20].

Ossowska et al. compared the efficacy of ANN with traditional techniques for diagnosing periodontitis by using variables like gender, age, use of nicotine, approximal plaque index, BOP, CAL and PD and found that no statistically significant differences in the assessment method for periodontal disease using ANN (sensitivity and specificity to diagnose a periodontitis patient was 85.7% and 80.0%, respectively) [32]. Shang et al. used intraoral photographs and U‐Net to detect no decayed teeth, degree of wear facets, presence of calculus and areas of gingival inflammation. A comparison between U‐Net and Deep‐Lab‐V3/PSP‐Net for image recognition showed that the U‐NET network improves the segmentation process by 10% with an average precision ranging from 50 to 60 [21]. Li et al. screened patients with gingivitis for calculus and soft deposits by using intraoral photographs using the Multi‐Task Learning CNN model. The author found that CNN is a cost‐effective method for diagnosing dental conditions and diseases. CNN was able to identify areas of gingival inflammation, calculus and soft deposits accurately [76]. Recently SVM, ANN and DT have also been tried to diagnose halitosis by measuring the concentrations of ‘methyl mercaptan’ in the breath as an indicator of halitosis [77]. AI‐app‐based can be used to detect halitosis by analysing the photographs of the tongue and estimate the degree of oral malodor. This can be a useful tool for screening patients with malodor before professional treatment and patient education for halitosis [77].

Yauney et al. by using a ‘fluorescent porphyrin biomarker for intraoral imaging’, along with ML and periodontal examination correlated the periodontal status with the systemic diseases. The periodontal disease status was cross‐correlated with a systemic condition (body mass index, neurological, cardiac rhythm, blood pressure and evaluation of the tympanic membrane and optic nerve). Gingivitis and periodontal disease were found to be associated with abnormalities in the optic nerve. A significant correlation between periodontitis with a family history of eye disease and swollen joint swellings was made by the model [25]. AI‐based models are also used for identifying genetic variants for periodontal disease. Ning et al. analysed periodontitis‐related transcriptomic datasets and immunosuppression genes by using ‘Dis‐Ge‐NET' and 'HisgAtlas’. The authors also identified differentially expressed genes, transcription factors and various signalling pathways, which are involved in periodontitis using bioinformatics analysis. The SVM classifier was used to identify three ‘master’ immunosuppression genes (FOS, FCGR3A, PECAM1). The following transcription factors: FOS, NFKB1, JUN, STAT4, STAT5B and HIF1A were also identified [58].

### 3.2 Role of AI‐Based Model for Analysis of Anatomical Landmarks (Cementoenamel Junction, Alveolar Bone Height and Tooth Wear)

AI, CNN and DL are also used for locating various anatomical landmarks and structures via radiographs. Some of the common structures and their associated defects that can be detected via AI include the type and morphology of crown and root of the teeth; amount of tooth wear; location of fracture lines; location of CEJ; alveolar bone height and degree of bone resorption/loss; amount of tooth mobility and soft tissue (gingival) profile [4, 7]. Al‐Haidan et al. predicted the severity of tooth loss by using ANN and utilising the following patients' data: age, smoking status, nature of brush and frequency of brushing, use of pickles, citric fruits, dried seeds and carbonated/acidic drinks and parafunctional habits such as bruxism. The study found that ANN can detect the severity of occlusal wear with an accuracy of more than 80% [8, 78].

Studies have reported that detection of bone levels/loss is difficult to detect by conventional radiographs, due to the presence of uneven illumination problems in dental radiographs and the complex and intricate anatomy of the alveolar bone. Hence, the use of AI‐based systems for the diagnosis of alveolar bone loss/level is often researched. Krois et al. analysed and compared the efficacy of CNN in diagnosing bone loss by using OPGs and compared it with the diagnosis made by six experts. The CNN model measured the percentage of bone loss in 2001 image segments from panoramic radiographs and showed a mean classification accuracy of (0.81 ± 0.02), which was more than that of the dentists (0.76 ± 0.06) (*p* = 0.067). The CNN achieved an impressive 81% accuracy for PBL detection. Additionally, the panoramic radiographs were manually cropped to focus on a single tooth and flipped vertically by 180° during pre‐processing. The results demonstrate that the diagnostic performance was validated in certain subgroups of teeth, with molars showing the highest accuracy (86%) [36]. AI models are also trained to identify cementoenamel junction, morphology of single‐rooted and multirooted teeth, the crest of the alveolar bone loss and check for variation based on the location of the teeth [44]. Lee et al. also reported that Deep CNNs can detect periodontally compromised teeth with a diagnostic accuracy of 98% compared to 75.5% for dentists [11]. A diagnostic accuracy of 76.7% for molars and 81% for premolar teeth with periodontal compromised condition were recorded. This was comparable to the results of 73% for the anterior teeth. The author stated that the supervised deep learning using a CNN‐based model called VGG‐16 (Visual Geometry Group) on a periapical radiographic dental dataset showed a highly accurate predictive capability, comparable to that of periodontists. The deep CNN algorithm was more accurate at distinguishing between no bone loss and bone loss in the upper and lower incisor teeth. The diagnostic accuracy for severe bone loss was the lowest overall, and the trained deep CNN algorithm was poorly optimised for the detection of severe bone loss. Further studies on the mechanisms underlying deep CNN algorithms are necessary. In 2022, a deep‐learning (DL) model was used to measure the bone level, and based on the bone loss, periodontal diagnosis and staging were compared between the dentist and AI model. No significant difference was noted for the level of bone loss measured by the DL and examiners (*p* = 0.65). The area under the receiver operating characteristics curve of RBL stage assignment for stages I, II and III was 0.89, 0.90 and 0.90, respectively. The accuracy of the case diagnosis was 0.85. The DL model was found to be effective in measuring bone loss measurements and image‐based periodontal diagnosis using periapical radiographic images. The accuracy of the model to detect bone loss was found to be 79.54% [43].

Huang et al. and Lin et al. also proposed an ‘automatic detection method (ABL‐ifBm) to detect bone loss based on the intensity and the texture of structure on the radiograph and found a sensitivity and specificity of the method to be only 92.5% and 14.0%, respectively [79, 80]. The same authors in 2011 compared the efficacy of ‘ABL‐ifBm’, ‘Two‐Stage Least Squares (TSLS) and bilateral filtering method for alveolar bone loss detection and found that ‘ABL‐ifBm’ is superior compared to another method for bone loss detection [81]. Lin et al. also tried to use ‘ABL‐ifBm’ and TSLS to determine the amount of bone loss by determining the location of the CEJ, apex of the root and crest of the alveolar bone. The study also compared the efficacy of ‘ABL‐ifBm’ and TSLS to a dentist and found that 53% of the CEJ could be localised with only 0.15 mm deviation from the positions marked by dentist. For alveolar bone loss, ABL‐ifBm showed a deviation of less than 10% compared to the dentist [81]. Nguyen et al. compared the diagnosis made by clinicians with different levels of experience and the ML model for alveolar bone loss. This was done by assessing the ability of the ML model to measure the distance from the CEJ to the alveolar bone crest. It was found that ML assessment of bone loss was strongly correlated with the diagnosis made by the clinician (*p* < 0.001) [82]. Other DL networks such as ‘HYNETS (hybrid network for periodontitis staging)’ and ‘Py‐Torch‐based (YOLO‐v5)’ are also being tried to assess periodontal bone loss and grade periodontitis severity [83]. The 'HYNETS model' can help the dentist to make a quicker periodontal diagnosis and automatically stage the diseases for faster treatment planning [30]. Dank et al. used a DNN to predict landmarks and evaluate the periodontal bone loss and severity and stage of periodontal disease in both single and multirooted teeth by using periapical radiographs. The authors found that DNN can localise the landmark with 83.3% accuracy for identifying the root morphologies. The DNN was found to have an error of 58% compared to the bone loss detected by dentists [42].

Alotaibi et al. and Ryu et al. developed a deep CNN algorithm to evaluate the accuracy of detecting bone loss around healthy and diseased teeth by analysing the periapical radiographs [84]. The accuracy of the system to diagnose alveolar bone levels for the binary classification (73.04%) was more compared to the multi‐classification (59.42%). The sensitivity and specificity of detecting diseased and healthy alveolar bone were 79.1% and 73%, respectively [84, 85].

Many recent studies have compared the efficacy of AI‐based methods for detecting alveolar bone loss and found AI tools to be accurate in diagnosing bone levels and staging periodontal disease. Jiang et al. demonstrated that AI is as capable as dentists when it comes to diagnosing vertical resorption and furcation lesion. The precision in detecting furcation and PBL for AI was 0.94 and the sensitivity was 0.75. For the vertical bone loss detection, the precision and specificity of the YOLO‐v4 model were 0.88 and 0.51, respectively. However, the accuracy values were lower for maxillary molars and mandibular anterior teeth. This indicates that overlapping anatomical structures may affect the diagnostic performance of the anterior region in panoramic radiographs [47].

Karacaoglu et al. employed deep‐learning models to detect radiographic bone level (or CEJ level) as a basic structure for automatically diagnosing bone loss and staging for periodontal disease and found that compared to the gold standard, the ability of both human observers and the ML to detect periodontal defects was significantly different (*p* = 0.05). However, ML and human observers exhibited similar performance in detecting periodontal defects with no significant differences (*p* = 0.05). This could help dentists with the diagnosis as well as the systematic and precise monitoring of periodontitis on panoramic X‐rays, diagnosis and systematically and precisely monitoring periodontitis on panoramic radiographs; thus, it could provide a significant improvement in dentists' ability to diagnose and treat periodontitis [86]. Shon et al. also reported an accuracy using U‐Net and YOLOv5 of 0.929 for assessing alveolar bone loss, planning for treatment and prevention based on early diagnosis, and the determination of the rate of disease progression. AI‐based diagnosis is deemed superior to manual or clinician‐based detection for diagnosing furcation defects. The model yielded satisfactory results for furcation lesions, while the results for vertical absorption were relatively low in specificity and high in accuracy [22]. Bayrakdar et al. assessed the accuracy of CNN: U‐Net architecture using panoramic OPG images for detecting vertical and horizontal bone loss, furcation defects and total alveolar bone loss. The author found the highest accuracy for detecting total alveolar bone loss and the lowest values for vertical bone loss, confirming that the CNN network is effective for the automatic determination of periodontal disease severity and treatment planning [45].

Hoss et al. compared the data of different types of CNN networks for the detection of PBL and found that all five CNNs, ResNet‐18 (ACC 82.8%; AUC 0.884), MobileNetV2 (82.0%; 0.884), ConvNeXT/s (83.9%; 0.903), ConvNeXT/b (84.8%; 0.911) and ConvNeXT/l (84.2%; 0.913) showed the same performance. The CNNs achieved an overall ACC between 82.0% and 4.8%. When considering the ability of the tested CNNs to detect PBL with sextants on periapical radiographs, differences between teeth in the lower and upper jaw were observed. Here, the projection technique and overlaying anatomical structures such as the maxillary sinuses or the nasal cavities may have negatively affected the diagnostic performance in the upper jaw. In contrast to the maxilla, mandibular sextants can be captured more accurately by the use of the right‐angle technique, which results in less distorted images and better diagnostic performance data [49]. Widyaningram et al. compared the Multi‐Label U‐Net and Mask R‐CNN for panoramic radiograph segmentation to detect periodontitis and found that Mask R‐CNN showed lower performance in image segmentation than Multi‐Label U‐Net. Mask R‐CNN could segment each object and distinguish between teeth for assessing the periodontitis stage on panoramic radiographs. Multi‐Label U‐Net outperformed Mask R‐CNN in image segmentation for periodontitis detection on panoramic radiographs. As a result, the identification of periodontitis was limited to blocks of teeth that were not specific to the individual level of RBL in the interdental alveolar crest [50]. Sunnetci et al. analysed 1432 images by an expert and extracted 1000 deep image features for each image using AlexNet and SqueezeNet deep‐learning architectures and found that the best classifiers for AlexNet‐based, SqueezeNet‐based and Direct‐Convolutional Neural Network (CNN) are Linear SVM, Medium Gaussian SVM and EfficientNetB5, respectively. The Linear SVM is the best classifier, with accuracy, error, sensitivity, specificity, precision and F1 score values of 81.49%, 18.51%, 84.57%, 79.14%, 75.68% and 79.88%, respectively [48, 51]. Kim et al. proposed a deep learning‐based method called DeNTNet to develop an automated diagnostic support system detecting bone loss due to periodontitis capable of panoramic dental X‐rays and found superior PBL detection performance compared to dentists [87]. Schulze et al. assessed the performance of manual versus the use of CNN for detecting lesions around the periodontium via cone beam computed tomography (CBCT). The extent of the angular defect (vertical bone loss), furcation defects and changes in the bone at the periapex was evaluated by a deep CNN and dentists. The CNNs showed significantly lower diagnostic accuracy compared to the dentist for detecting the lesions. The clinician achieved better results, except for the diagnosis of vertical/angular defects [88].

AI models are also being used to assess the degree of soft tissue (gums around teeth). Yang et al. 2023 in a pilot animal study used three‐dimensional gingival soft tissue reconstruction images and cone beam computed tomography to determine the nature of the gingival phenotype. The gingival phenotype (thickness) was clinically assessed via a periodontal probe and digital calliper and compared with the digital estimation by DL‐based CBCT. A positive correlation between the measurements made by the dentists and the DL model was noted. There was no difference in the agreement between the virtual and clinical measurements on sexually matured samples (0.087 ± 0.240 mm) and pre‐pubertal samples (0.033 ± 0.195 mm). Noticeably, there is a greater agreement between the virtual and clinical measurements at the buccal sites (0.019 ± 0.233 mm) than at the lingual sites (0.116 ± 0.215 mm) [33]. Chifor et al. also tried to use 3‐dimensional ultrasound for periodontal tissue (pocket depth and gingival tissue thickness) analysis by using ‘RR‐CNN and U‐Net‐CNN models. CNN could effectively detect both the depth of the periodontal pocket (10%) and gingiva thickness (75.6%) and this ability to measure the PD was linked to two techniques (wavelet transforms and pattern classification) [89, 90]. Studies have shown that with pattern classification, 86.6% of the PD can be predicted effectively and AI‐assisted periodontal probing provides faster and more reliable pocket charting for dentists. The 5th generation periodontal probe that uses ultrasonic waves to detect PD is also based on the pattern and image classification system of AI [91, 92]. A recent randomised clinical trial compared the efficacy of a manual periodontal probe to an AI‐based periodontal probe (PA‐ON Parometer, Germany) and found that the mean PD varied significantly between manual and PA‐ON probe (mean difference: 0.38 mm (*p* < 0.001)). The time required for the PA‐ON probe was longer (23 ± 11 min) compared to the manual probe (21 ± 11 min) with a *p*‐value < 0.05. The pain score showed statistically significant less for the PA‐ON probe compared to the manual probe [55].

### 3.3 Role of AI in Identifying the Type of the Implant System, Implant Prosthesis and Peri‐Implant Health

AI and its various subsets are also used to detect the nature and type of implant; type of implant thread morphology; brand of the dental implant and its prosthesis; degree of osseointegration and bone loss around the implant. Sukegawa et al. used OPG images to diagnose and classify different dental implant brands by using different types of deep CNN models (VGG‐16, VGG‐19 and finely tuned VGG‐16 and VGG‐19). Among these, the finely tuned VGG‐16 model was found to have the highest efficacy in identifying the implant followed by finely tuned VGG‐19 and normal transfer‐learning VGG‐16 [54]. A multi‐task DL model can also be tried to identify the type of implant system and suggest the most appropiate treatment plan. Lee et al. used image pre‐processing and transfer‐learning techniques based on fine‐tuned and pre‐trained deep CNN architecture (GoogLeNet V3) to assess and compare the accuracy, sensitivity and specificity between CNN and a periodontist for identifying dental implants via OPGs. The authors found that the deep CNN can detect and classify the implant system as accurately as the periodontist [93, 94]. Lee et al. compared the accuracy of classifying various dental implant systems using OPGs and found that the DL algorithm significantly improved the diagnosis (78.88%) compared to no use of DL (63.13%). The board‐certified periodontists using the DL algorithm have a mean accuracy (8.56%) compared to the use of the DL algorithm alone. However, if dentists are not specialised in implantology, the mean accuracy of DL is better than dentists for diagnosing implant systems [61, 95, 96]. Similar results were obtained by a study by Park et al. 2023 where the DL showed lower mean accuracy (23.5% ± 18.5) compared to an implantologist (43.3% ± 20.4%), but more than a non‐specialised dentist (16.8% ± 9.0%). The time taken by the DL models to read and classify was less (4.5 min) compared to the implantologist (75.6 ± 31.0 min) and non‐specialised dentists (91.3 ± 38.3 min) [35, 97]. A recent systematic review evaluating the role of DL in identifying and classifying dental implant systems from dental imaging showed that DL‐based implant classification accuracy ranged from 70.75% to 98.19%. However, most of the studies included were judged to have a high risk of bias due to inappropriate methods of data selection and reference standards [35]. Kim et al. evaluated 355 implant fixtures from three implant systems (Osstem, Dentunum and Straumann) using a deep CNN and found that ‘YOLOv3’ is the best in classifying the Straumann dental implant system [52, 98]. Jang et al. have tried the DL model and Faster R‐CNN to detect the peri‐implant tissues using periapical radiographs [89]. AI models are also used for identifying implant fractures. Lee et al. assessed three different types of deep CNN models (VGGNet‐19, Deep CNN and GoogLeNet Inception‐v3) for the detection of fractures in dental implants. A total of 21 398 dental implants were reviewed and it was found that all three Deep CNN models can detect fractured implants with an accuracy of over 0.80. The automated Deep CNN showed the highest and most reliable detection method as compared to a GoogLeNet or finely trained VGGNet‐19 models [57, 99].

The detection of the marginal bone level around implants by radiographs is often difficult as the margins of the marginal bone surrounding implants make the area blurred and difficult to diagnose. The identification of buccal and lingual bone heights along with hard and soft tissue profile assessment is difficult with conventional radiographic imaging. Therefore, the use of AI to evaluate the degree of bone loss around implants for the diagnosis of peri‐implantitis compared to conventional methods is being tried. Many recent studies have compared the use of AI for peri‐implant bone loss assessment compared to dentists. Vera et al. developed an image‐processing method using DL techniques to help dental professionals in defining the severity of marginal bone loss around implants. A DL model (YOLOv3) was used to identify the prosthesis and implant along with the changes in the intensity of bone and intersections between the screw and crown. The authors found that the DL model could detect bone loss around implants with less deviation in pixels [64]. CNN models are also found to be accurate in detecting bone loss around implants with not much difference in the diagnostic accuracy between the AI system and the expert [60]. A study by Cha et al. also reported that the R‐CNN model can detect landmarks around dental implants and help assess the severity of peri‐implantitis, without any statistically significant difference between the modified R‐CNN model and dental clinicians [100]. Wang et al. investigated a group of peri‐implantitis patients who were planned to undergo regenerative therapy and performed a comprehensive clinical examination along with immunological and microbial profiling using an ML machine‐learning model for checking the immune profile. The results showed that an unsupervised ML model can identify individuals with distinct microbial colonisation and immune profiles, and predict the outcomes of the regenerative therapy. Individuals with low‐risk exhibit lower B‐cell infiltration and increased levels of M1 to M2 macrophage ratios. The immune profile for low‐risk individuals also showed an increase in levels of complement and Th1/Th17 cytokines. The levels of two periodontal pathogens *Prevotella intermedia and Fusobacterium nucleatum* were also found to increase in individuals with high risk. Hence, ML model can be used to predict the signatures immune and microbiological markers and this could be useful for predicting peri‐implantitis [59].

### 3.4 AI in Periodontal/Peri‐Implant Disease Risk Prediction/Determination of Prognosis

Prediction of prognosis before and after any therapy is an important parameter to determine the success rate of the treatment. Many AI‐based models and apps have been developed to determine patients' overall prognosis and monitor the treatment progress by considering tooth‐related and patient‐related risk factors. Previously, many computerised risk assessment tools were used to assess periodontal disease prognosis [101]. For example, a computerised tool referred to as the ‘Periodontal‐Risk‐Calculator (PRC)’, was used to predict the risk for initiation of periodontal disease in healthy patients, the degree of periodontal deterioration and the risk of disease progression; and generate treatment options for guide the clinician [34, 102]. The calculation for PRC was based on a mathematically based algorithm that involves many patient‐related factors such as the age of the patient; any history of diabetes, smoking, previous periodontal surgery; PD, presence or absence of furcation involvements; number of restorations; degree of subgingival calculus; radiographic bone levels and amount of vertical bone loss. Studies have found PRC to be effective in reducing the disease burden and improving the overall health and cost of dental treatment. PreViser Corporation improved the original PRC tool and launched a new software called ‘Oral Health Information Suite’ for periodontal‐risk assessment [103, 104]. Patel et al. created a ‘Perio‐Risk Scoring system’ (PRSS) using a decision support system to guide clinicians in generating periodontal scores and identifying the risk factors in patients. The authors compared the similarity between the scores given by all the five risk assessment methods [PRSS, PreViser, periodontal‐risk assessment (PRA), Cigna, Sonicare] on 20 patients and found PRSS to be the most accurate in predicting the risk (70%). The risk prediction by other methods was found to be as follows: Cigna (25%), PRA (35%), PreViser (55%), Phillips (35%) and [103]. Recently ‘The Internet of Things (IoT)’, a new technology that uses a system of various inter‐connected gadgets and AI was used to provide preventive oral care. Studies have found a positive response to using IoDT in preventive oral care, in‐home dental healthcare and for diagnosing various oral diseases/conditions [104, 105]. An IoT‐based neural network can be used to identify tooth structure, gaps between teeth, caries and dental biofilm, diagnose gingival and periodontal disease, and detect displacement of the implant [38].

‘Deetal‐Perio’ is another region‐based CNN tool for predicting the severity of periodontitis, and this AI‐based model could effectively assist the dentist in diagnosis and disease prediction for better patient management [106]. Patel et al. in 2022 and 2023 developed and tested another periodontal disease prediction model using ML (XGBoost) for predicting gingival phenotypes [107, 108]. This ML model used an algorithm based on the number of follow‐ups and dental visits by the patients. Recently, AI‐based models have also been used to check the compliance of patients and monitor their oral hygiene status [108, 109]. Balaei et al. proposed two algorithms by examining the intraoral photographs of patients before and after periodontal therapy. The subjects before and after the treatment were considered diseased and healthy individuals. Using pixel intensity as the feature to classify the images into healthy and diseased sites, the system showed to have 66.7% accuracy. These algorithms could also identify the improvement after the treatment with an accuracy of 91.6%. These algorithms can be connected to the smartphone and can be used for monitoring individuals where access to dental care is limited. The algorithm is also useful for personnel who are not specialised in dentistry to monitor their dental health and the progress of their oral hygiene after treatment [109, 110]. Studies have found that when AI‐based monitoring for patients is done, patients show better outcomes after treatment compared to those who were given oral hygiene instruction in the dental office alone [40]. Shimpi et al. proposed a new periodontal disease risk assessment model using supervised ML methods, which can be used at the point of care. The authors compared the performance of SVM, ANN, DT, Naïve Bayes and Logistic Regression (LR) and found that ANN and DT were better at classifying patients with risk of periodontitis compared to SVM, NB and LR [111].

AI‐based tools are also used to determine the outcome of implant treatment and predict the risk of developing peri‐implantitis. Mameno et al. predicted the risk of peri‐implant diseases by using LR, SVM and RF by using data from patients related to patient‐related risk factors. The study evaluated 127 implants with and without peri‐implantitis and found that the RF model has the highest ability to predict the onset of peri‐implantitis with an accuracy of 0.70. The study also found that the duration of implant in function and the oral hygiene status of the patient are two of the most vital determinants for predicting the risks associated with the development of peri‐implantitis. Plaque control records, history of smoking, less than 2 mm width of keratinized mucosa and lack of occlusal supports are some of the important factors that should be considered while assessing the risk of peri‐implantitis using AI models [56]. Cetiner et al. also tried to predict the health of the peri‐implant mucosa using three different types of AI models. The implants were classified based on the health of peri‐implant mucosa into three types: peri‐implant health, peri‐implant mucositis and peri‐implantitis. Both clinical and risk factor analyses were used to predict the prognosis of the peri‐implant disease. Different AI methods such as DT; J‐48, LR, ANN, and multilayer perceptron [MLP] were compared to predict the health of peri‐implant tissue. The study found that the ‘J48 method’ has the highest prediction accuracy compared to logistic regression and MLP methods. BOP, supportive maintenance treatment/recall and nature of medication were key parameters for predicting the health of the peri‐implant tissues [62]. Rekawek et al. also found that the RF model has the highest predictive performance for dental implant failures and peri‐implantitis among Logistic regression, RF classifiers, SVM and ensemble techniques. The rate of implant failure can be correlated to the amount of local anaesthetic used, length and diameter of the implant, preoperative use of antimicrobials, number of dental visits, and history of diabetes mellitus [34]. Studies have also assessed the outcome of the implant treatment based on the marginal bone loss by using DL and degree of osseointegration [112]. Oh et al. assessed OPGs and periapical radiographs of 580 patients with 1206 dental implants and used seven different DL models to predict the amount of implant stability and bone formed around the implant (indicating the level osseointegration). The study found that DL can assess the degree of osseointegration (the range for accuracy, sensitivity and specificity was found to be 0.799–0.836; 0.811–0.833 and 0.780–0.857, respectively). This could help the dentist assess the success rate, identify the failure at an early stage and plan the implant treatment more effectively and quickly [46, 63].

## 4 Conclusion

AI and its various subsets are used in various aspects of periodontology and implantology. AI‐based tools such as DL, ML, CNN and ANN are being tried for more effective, quicker and timely diagnosis, risk assessment and treatment plans for periodontal and peri‐implant disease diagnosis. Among all these tools, DL and CNN are the most commonly used tools for the diagnosis of bone levels around teeth or implants, periodontal disease staging and severity and location of anatomical structures and teeth. These AI‐based tools can be used as educational guides for training future dentists and help general dentists treat and diagnose oral diseases and conditions. However, one should note that these AI‐based tools should only be used as adjuncts; no machine should replace the intellect of the human mind, and the humane touch is the most essential part of any patient care in healthcare settings.

## Author Contributions

A.C. and R.R.: concept/design; A.C. and R.R.: data analysis/interpretation; A.C., R.R., S.K., S.G.B.: drafting article; S.G.B.: critical revision of article; A.C., R.R., S.K., S.G.B.: approval of article; A.C. and S.K.: tables.

## Ethics Statement

The authors have nothing to report.

## Consent

The authors have nothing to report.

## Conflicts of Interest

The authors declare no conflicts of interest.

## Peer Review

The peer review history for this article is available at https://www.webofscience.com/api/gateway/wos/peer‐review/10.1111/joor.14045.

## Supporting information


Table S1.

## Data Availability

The data can be shared upon reasonable request from the corresponding author via email.
