# Supplementary material for: Applications of Artificial Intelligence (AI) for Diagnosis of Periodontal/Peri‐Implant Diseases: A Narrative Review
Source: J Oral Rehabil. 2025 Jun 4;52(8):1193–219. doi: 10.1111/joor.14045 (PMC12392392; doi:10.1111/joor.14045)
Supplement: Supplementary file 1 — Table S1. [file JOOR-52-1193-s001.doc]

| **Table 1: Search Strategy and number of articles retrieved from different databases** | | | |
| --- | --- | --- | --- |
| **S/No** | **Database** | **Search string used** | **No of articles** |
|  | **PubMed** | ("periodontal"[Title/Abstract] OR "periodontal disease"[Title/Abstract] OR "periodontitis"[Title/Abstract] OR "periodontal defect"[Title/Abstract] OR "periimplantitis"[Title/Abstract] OR "periimplant disease"[Title/Abstract]) AND ("Artificial intelligence"[Title/Abstract] OR "Machine learning"[Title/Abstract] OR "Deep learning"[Title/Abstract] OR "Convolutional neural network"[Title/Abstract] OR "Artificial Neural network"[Title/Abstract]) | **308** |
|  | **Scopus** | "periodontal" OR "periodontal disease" OR "periodontitis" OR "periodontal defect" OR "periimplantitis" OR "periimplant disease" AND "Artificial intelligence" OR "Machine learning" OR "Deep learning" OR "Convolutional neural network" OR "Artificial Neural network" | **566** |
|  | **Web of Science** | **((TI=("Artificial intelligence" OR "Machine learning" OR "Deep learning" OR "Convolutional neural network" OR "Artificial Neural network")) AND TI=("periodontal" OR "periodontal disease" OR "periodontitis" OR "periodontal defect" OR "periimplantitis" OR "periimplant disease"))** | **93** |
|  | **Preprint/ conference proceedings (IEEE explorer** | "Artificial intelligence" OR "Machine learning" OR "Deep learning" OR "Convolutional neural network" OR "Artificial Neural network" AND "periodontal" OR "periodontal disease" OR "periodontitis" OR "periodontal defect" OR "periimplantitis" OR "periimplant disease" | **52** |
|  |  | **Total Search** | **1019** |
